# Doxycycline inhibits leukemic cell migration via inhibition of matrix metalloproteinases and phosphorylation of focal adhesion kinase

**DOI:** 10.3892/mmr.2015.3833

**Published:** 2015-05-25

**Authors:** CHUNHUAI WANG, RU XIANG, XIANGZHONG ZHANG, YUNXIAN CHEN

**Affiliations:** 1 Department of Hematology, The First Affiliated Hospital of Sun Yat-Sen University, Guangzhou, Guangdong 510080, P.R. China; 2 Department of Hematology, The Second Affiliated Hospital of Anhui Medical University, Hefei, Anhui 230601, P.R. China; 3 Department of Internal Medicine, School of Nursing, Anhui Medical University, Hefei, Anhui 230601, P.R. China

**Keywords:** doxycycline, leukemic cells, migration, focal adhesion kinase, gelatinases

## Abstract

Doxycycline, a tetracycline-based antibiotic, has been reported to attenuate melanoma cell migration through inhibiting the focal adhesion kinase (FAK) signaling pathway. However, it remains to be elucidated whether doxycycline exerts this effect on leukemia cell migration. The present study aimed to examine the role of doxycycline in leukemia cell migration. The invasion capacities of the human leukemia cell lines KG1a (acute myelogenous leukemia) and K562 (chronic myelogenous leukemia) were evaluated using Matrigel^®^ matrix-coated Transwell^®^ chamber assays; leukemic cell lines treated with doxycycline (1 *µ*g/ml) or anti-β1-integrin antibodies were added to the upper chamber, while untreated cells were included as controls. Reverse transcription quantitative polymerase chain reaction was performed in order to further understand the influence of doxycycline treatment on the expression of FAK and gelatinases in the KG1a and K562 leukemic cell lines. In addition, FAK protein expression and phosphorylation were determined using western blot analysis in order to investigate the mechanism by which doxycycline inhibited leukemic cell migration. The results revealed that doxycycline treatment significantly attenuated the migration of KG1a and K562 cells, which was demonstrated to be associated with inhibition of the expression and phosphorylation of FAK. In addition, doxycycline treatment inhibited matrix metalloproteinase (MMP)-2 and MMP-9 expression. Furthermore, incubation with blocking anti-β1-integrin antibodies had an analogous inhibitory effect on leukemic cell migration to that of doxycycline. In conclusion, the results of the present study suggested that doxycycline attenuated leukemic cell migration through inhibiting the FAK signaling pathway. Therefore, doxycycline may have potential for use as a novel strategy for the treatment of leukemia.

## Introduction

The migration of cancer cells is a key step in tumor metastasis, which is associated with high mortality rates in cancer (1). The metastatic process involves the movement of cancer cells through the extracellular matrix (ECM) around the region of the primary tumor and requires adhesion of cancer cells to the ECM as well as ECM degradation (2). Focal adhesion kinase (FAK) and matrix metalloproteinases (MMPs) have been regarded as critical molecules in this process and previous studies indicated that the FAK pathway indirectly influences MMP activity as well as cell-ECM interactions (3–5).

Doxycycline, a member of the tetracycline group of antibiotics, is commonly used to treat a variety of infections (6). Numerous studies have demonstrated that doxycycline induced tumor apoptosis and suppressed tumor cell migration (7–9). Furthermore, the role of doxycycline as a non-specific MMP inhibitor has been established (10). Previous studies have demonstrated that doxycycline inhibited solid tumor metastasis via downregulation of FAK (11,12). However, it remains to be elucidated whether doxycycline has an analogous effect on leukemia cells.

Acute myelogenous leukemia (AML) is a hematological malignancy, which may be regarded as a prototype of metastatic cancer. AML is characterized by the premature egress of leukemic blasts from the bone marrow and their dissemination into peripheral tissues (13). Increased gelatinase (MMP-2 and MMP-9) expression by AML blasts has been implicated in the invasive phenotype of AML (14). Expression of FAK in AML has been associated with enhanced blast migration, increased cellularity and poor prognosis (15).

The current study aimed to investigate the potential of doxycycline to attenuate the migration of leukemic cells through inhibiting leukemic cell expression of the gelatinases MMP-2 and MMP-9 via the FAK pathway. This study may therefore provide novel insights into the importance of doxycycline as a candidate for the treatment of leukemia patients.

## Materials and methods

### Chemical reagents

Doxycycline was purchased from Sigma-Aldrich (St. Louis, MO, USA). A stock solution was prepared at 10 mg/ml in phosphate buffered saline (PBS; Dingguo Biotech Corp., Guangzhou, China) and stored at −20°C.

### Cell culture

The human leukemia cell lines KG1a (acute myelogenous leukemia) and K562 (chronic myelogenous leukemia) were obtained from the Institute of Hematology and Hospital of Blood Diseases, Chinese Academy of Medical Sciences (Tianjin, China). Cells were cultured in RPMI 1640 medium (Gibco-BRL, Paisley, UK) supplemented with 10% fetal bovine serum (FBS; Gibco-BRL) and antibiotics (100 U/ml penicillin and 100 µg/ml streptomycin; Gibco-BRL). Cells were maintained at 37°C in humidified atmosphere containing 5% CO_2_.

### In vitro invasion assay

The invasion capacity of leukemic cells was evaluated using a Matrigel^®^-coated Transwell^®^ chamber system. RPMI 1640 medium (500 *µ*l supplemented with 10% FBS) containing 100 ng/ml stromal cell-derived factor-1α (Wako Pure Chemical Industries, Ltd., Tokyo, Japan), a chemoattractant, was added to the lower chambers in the 24-well Transwell^®^ plates (Corning, Inc., Corning, NY, USA). Membrane filters (Merck Millipore, Billerica, MA, USA; diameter, 6.5 mm; pore size, 8 *µ*m) were coated with 50 *µ*g Matrigel^®^ (BD Biosciences, San Jose, CA, USA), providing a composition similar to that of human basement membranes. Leukemic cell lines treated with doxycycline (1 *µ*g/ml) or anti-β1-integrin antibodies (100 ng/ml; Santa Cruz Biotechnology, Inc., Dallas, TX, USA,) were added to the upper chamber (2.0×10^5^ cells/well in 200 *µ*l RPMI 1640), as previously described (16). Untreated cells were included as controls. Transwell^®^ plates were incubated for 24 h at 37°C in a CO_2_ incubator. Subsequently, cells that had crossed the Matrigel^®^ and migrated to the lower surface of the filter were fixed, stained with 0.1% crystal violet (Dingguo Biotech Corp.) and enumerated in ten randomly selected fields per filter under a light microscope (Olympus CKX41-A32RC inverted microscope; magnification, ×200; Olympus Corp., Tokyo, Japan). The invasive cells on the lower surface of the membrane were stained by dipping inserts in the 0.1% crystal violet solution for 30 min. The inserts were then rinsed in water and allowed to air dry. Each invasion experiment was performed in triplicate.

### Reverse transcription quantitative polymerase chain reaction (RT-qPCR)

RT-qPCR was used to quantify messenger (m) RNA levels. KG-1a and K562 cells were treated with 1 µg/ml doxycycline (Sigma-Aldrich) or 100 ng/ml anti-β1-integrin antibody (anti-β1-integrin-Ab) at 37°C in a CO_2_ incubator for 24 h. A total of 1×10^6^ cells were placed in 1.5 ml tubes which were cooled on ice. Total RNA was extracted from cells using TRIzol^®^ reagent (Invitrogen Life Technologies, Foster City, CA, USA) according to the manufacturer's instructions. A total of 4 *µ*l RNA was used to perform RT using an ABI 7500 Real-Time-PCR system (Applied Biosystems, Foster City, CA, USA) with the SYBR Green master mix (Applied Biosystems) and primers (Da′an Gene Co., Guangzhou, China). The sequences of the primers used for qPCR analysis were as follows: human MMP-2 forward, 5′-GGC CCCACA GGA GGA GAA-3′ and reverse, 5′-GGT GCT GGC TGA GTA GAT CCA-3′; human MMP-9 forward, 5′-AGA TGC GTG GAG AGT CGA AATC-3′ and reverse, 5′-GTC TCG GGC AGG GAC AGTT-3′; human FAK forward, 5′-AGC AAG AAG AGC GCA TGAGG-3′ and reverse, 5′-GGG CGG TGC TTC ATC AGA-3′; human β-actin forward, 5′-GCA TGG GTC AGA AGG ATT CCT-3′ and reverse, 5′-TCG TCC CAG TTG GTG ACGAT-3′. The reaction conditions were as follows: 93°C for 3 min, followed by 40 cycles of 93°C for 30 sec and 55°C for 45 sec. β-actin was used as a reference to obtain the relative fold change for targets using the comparative Ct method (17). All samples were analyzed in triplicate.

### Western blot analysis

In one treatment set, KG1a and K562 cells were treated with doxycycline at 0.1 or 1 *µ*g/ml for 1, 3, 6 and 12 h. In another treatment set, KG1a and K562 cells were treated with 1 *µ*g/ml doxycycline or 100 ng/ml anti-β1-integrin-Ab for 24 h at 37°C in a CO_2_ incubator. Total protein was extracted from control leukemic cells and treated leukemic cells. Protein samples (40 *µ*g) were subjected to 10% SDS-PAGE (Dingguo Biotech Corp) and transferred onto polyvinylidene difluoride membranes (Merck Millipore). Membranes were then blocked with 5% skimmed milk in Tris-buffered saline with Tween 20 and reacted with the following primary antibodies: Anti-FAK (rabbit polyclonal; cat. no. 3283; 1:1,000 dilution), anti-p-FAK (Tyr576/577; rabbit polyclonal; cat. no. 3281; 1:1,000 dilution), anti-p-FAK (Tyr925; rabbit polyclonal; cat. no. 3284; 1:1,000 dilution), anti-MMP2 (rabbit polyclonal; cat. no. 4022; 1:1,000 dilution) and anti-MMP9 (rabbit polyclonal; cat. no. 2270; 1:1,000 dilution), which were purchased from Cell Signaling Technology (Danvers, MA, USA) as well as anti-p-FAK (Tyr397; rabbit monoclonal; cat. no. 44-625G; 1:1,000 dilution; Invitrogen Life Technologies). Following washing with PBS three times, membranes were incubated with goat anti-rabbit IgG, horseradish peroxidase-conjugated secondary antibody (Cell Signaling Technology, Danvers, MA, USA; cat. no. 7074; 1:1,000 dilution). Bands were visualized using enhanced chemiluminescence (SuperSignal West Pico chemiluminescent substrate; Pierce Biotechnology, Inc., Rockford, IL, USA). GAPDH (rabbit monoclonal; cat. no. 5174; 1:1,000 dilution; Cell Signaling Technology) was used as internal control and was detected on the same membrane.

### Statistical analysis

Values are expressed as the mean ± standard deviation, unless otherwise stated. Statistical significance was evaluated using one-way analysis of variance tests with SPSS 11.0 software (SPSS, Inc., Chicago, IL, USA). P<0.05 was considered to indicate a statistically significant difference between values.

## Results

### Effect of doxycycline and anti-β1-integrin on leukemic cell migration

The inhibitory effects of doxycycline and anti-β1-integrin-Ab blocking treatment on the invasion capacity of KG1a and K562 cells were investigated using Matrigel^®^ matrix-coated Transwell^®^ chamber assays. As shown in Table I, doxycycline and anti-β1-integrin-Ab were demonstrated to significantly decrease the number of migrated KG1a (P<0.001) and K562 (P<0.001) cells compared with that of the control group. This therefore indicated a marked reduction in the invasion capacity of these cells.

### Transcription of FAK and gelatinases in leukemic cells following doxycycline or anti-β1-integrin-Ab treatment

**Figure 1:**
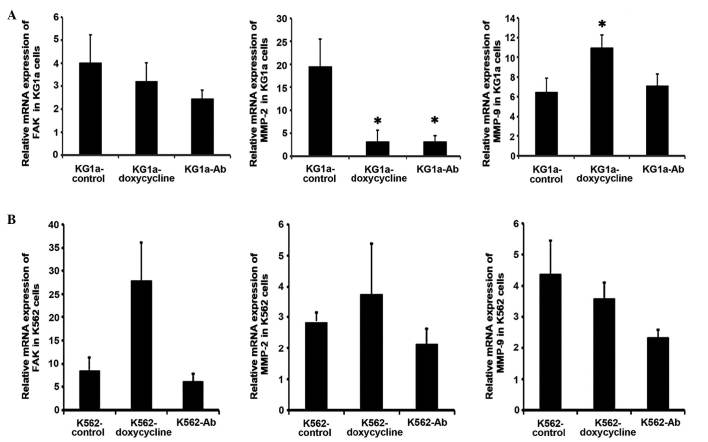
Effects of doxycycline or anti-β1-integrin-Ab treatment on the transcription of FAK and gelatinases (MMP-2 and MMP-9) in KG1a cells and K562 human leukemia cells. mRNA levels of FAK, MMP-2 and MMP-9 were analyzed using reverse transcription quantitative polymerase chain reaction in (A) KG1a cells and (B) K562 cells following treatment with doxycycline (1 *µ*g/ml) or anti-β1-integrin-Ab (100 ng/ml). mRNA expression was quantified relative to that of β-actin. Values are presented as the mean ± standard deviation of triplicate measurements. ^*^P<0.05 vs. control group. Ab, antibodies; FAK, focal adhesion kinase; MMP, matrix metalloproteinase; mRNA, messenger RNA.

RT-qPCR was conducted in order to further understand the influence of doxycycline or anti-β1-integrin-Ab treatment on the expression of FAK and gelatinases in the KG1a and K562 leukemic cell lines (Fig. 1A and B).

As shown in Fig. 1A, in KG1a cells, the levels of FAK mRNA were not significantly altered by doxycycline or anti-β1-integrin-Ab treatment compared with those of untreated control cells. By contrast, MMP-2 mRNA expression was significantly decreased following treatment with doxycycline or anti-β1-integrin-Ab compared with the control group (P<0.05). Furthermore, MMP-9 mRNA expression was significantly increased by doxycycline (P<0.05), whereas anti-β1-integrin-Ab treatment exhibited no significant effect. As shown in Fig. 1B, no significant changes were detected in the levels of FAK, MMP-2 and MMP-9 mRNA in K562 cells following treatment with doxycycline or anti-β1-integrin-Ab.

### Effects of different concentrations of doxycycline on FAK protein expression and phosphorylation

In order to investigate the mechanism by which doxycycline inhibited leukemic cell migration, FAK protein expression and phosphorylation were evaluated using western blot analysis. KG1a and K562 cells were treated with doxycycline at 0.1 or 1 *µ*g/ml for 1, 3, 6 and 12 h. FAK is known to undergo adhesion-dependent phos-phorylation on six tyrosine residues: 397, 407, 576, 577, 861 and 925 (12); however, since neither the Tyr-407 or Tyr-861 site has been reported to function in mediating interactions with effecter molecules, the functional significance of these sites remains uncertain (18). Therefore, Tyr397, Tyr576/577 and Tyr 925 were analyzed in the present study.

**Figure 2:**
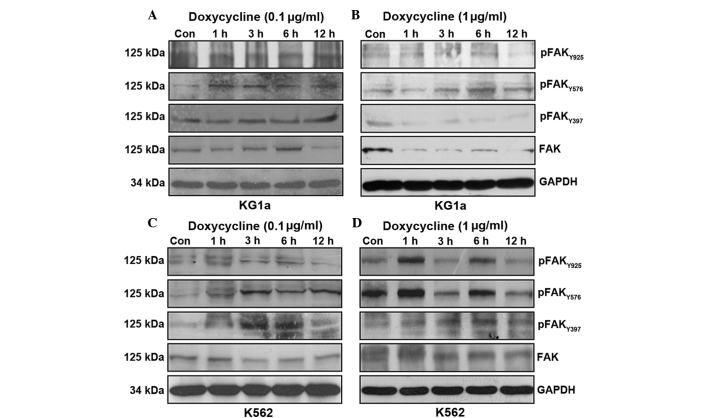
Dose- and time-dependent effects of doxycycline treatment on FAK protein expression and phosphorylation in KG1a and K562 leukemic cell lines. Levels of FAK protein and phosphorylated FAK at different sites (Y397, Y925 and Y576/577) were determined using western blot analysis following incubation with different doses of doxycycline for 1, 3, 6 and 12 h. (A) 0.1 µg/ml and (B) 1 µg/ml doxycycline treatment in KG1a cells. (C) 0.1 *µ*g/ml and (D) 1 µg/ml doxy-cycline treatment in K562 cells. GAPDH was used as the loading control. FAK, focal adhesion kinase; Y, tyrosine; Con, control; p, phosphorylated.

The effects of doxycycline on FAK protein expression and phosphorylation were not consistent in the leukemic cells. Exposure of KG1a cells to 0.1 *µ*g/ml doxycycline decreased only the total protein expression of FAK and this effect was only apparent following 12 h of treatment (Fig. 2A). Of note, following treatment of KG1a cells with 1 *µ*g/ml doxycycline, decreased total FAK protein expression and Tyr397 phosphorylation were observed at 1 h post treatment; in addition, Tyr925 phosphorylation was inhibited following 12 h of treatment (Fig. 2B). Doxycycline treatment had no significant effects on Tyr576/566 phosphorylation at either dose (Fig. 2A and B).

By contrast, exposure of K562 cells to 0.1 *µ*g/ml doxycycline revealed a time-dependent decrease in Tyr925 phosphorylation (Fig. 2C). However, an increased concentration (1 *µ*g/ml) of doxycycline exhibited no effect on total FAK expression and Tyr397 phosphorylation, although downregulation of Tyr576/577 and Tyr925 phosphorylation occurred following 12 h of treatment with 1 *µ*g/ml doxycycline (Fig. 2D).

### Expression of FAK, pFAK, gelatinases MMP-2 and MMP-9 following doxycycline or anti-β1-integrin-Ab treatment

**Figure 3:**
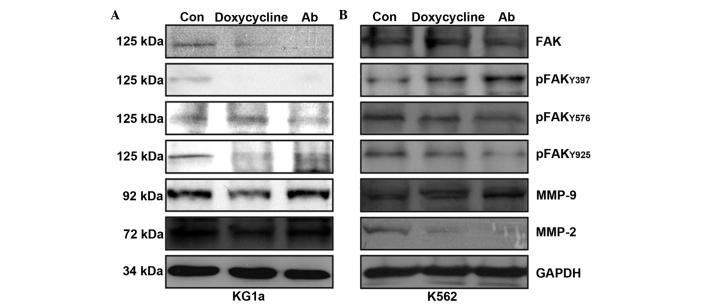
Effects of anti-β1-integrin-Ab blocking treatment on FAK protein expression and phosphorylation as well as the expression of gelatinases (MMP-2 and MMP-9) in KG1a and K562 leukemic cell lines. Protein expression levels of FAK, pFAK at sites (Y397, Y925 and Y576/577), MMP-2 and MMP-9 were determined using western blot analysis in (A) KG1a and (B) K562 cells treated with doxycycline (1 µg/ml) or anti-β1-integrin-Ab (100 ng/ml). GAPDH was used as the loading control. Ab, antibodies; FAK, focal adhesion kinase; MMP, matrix metalloproteinase; Y, tyrosine; Con, control; p, phosphorylated.

In order to investigate whether signaling downstream of the FAK pathway was involved in the doxycycline- or anti-β1-integrin-Ab-mediated downregulation of gelatinases, KG1a and K562 cells were treated with doxycycline (1 *µ*g/ml) or anti-β1-integrin-Ab (100 ng/ml) for 24 h. As shown in Fig. 3A, the expression of MMP-2, FAK, Tyr397-p-FAK and Tyr925-p-FAK were potently decreased by doxycycline and anti-β1-integrin-Ab treatment in KG1a cells. The anti-β1-integrin-Ab also inhibited the expression of MMP-9 in KG1a cells. As shown in Fig. 3B, both doxycycline and anti-β1-integrin-Ab inhibited MMP-2, Tyr576/577-p-FAK and Tyr925-p-FAK in K562 cells, while FAK, Tyr397-p-FAK and MMP-9 were not impacted. However, exposure of K562 cells to identical conditions decreased only MMP-9 protein expression following doxycycline treatment (Fig. 3B).

## Discussion

Tetracycline is a polyketide, which is produced by the *Streptomyces* genus of Actinobacteria and has been used as a broad-spectrum antibiotic for decades (19). Tetracycline functions as a protein synthesis inhibitor by binding to the 16S ribosomal RNA portion of the 30S ribosomal subunit and preventing amino-acyl transfer RNA from binding to the ribosome (20). Doxycycline, a member of the tetracycline group of antibiotics, has been reported to have a variety of antitumor effects *in vitro* (21), including impairment of mitochondrial protein synthesis (22,23), proliferation arrest in the G_1_ phase of the cell cycle (24) and induction of apoptosis via caspase-3 activation (8). The present study confirmed that doxycycline (1 µg/ml) exerted inhibitory effects on the proliferation of leukemia cells, with no significant cytotoxic effects detected using cell counting kit-8 assays *in vitro* (data not shown).

Studies have demonstrated that doxycycline exhibited direct weak cytotoxic and indirect inhibitory effects on tumor cell proliferation, angiogenesis, metastasis and migration through multiple targets (11,25,26). However, the molecular mechanism of the antitumor effects of doxycycline remains to be fully elucidated. It was speculated that the interaction between tumor cells and ECM may be a critical stage in this process, leading to a series of consequential biological actions that control important tumor cell phenotypes (27,28). The *FAK* gene is ubiquitously expressed and encodes a non-receptor tyrosine kinase that localizes to focal adhesions on the cell membrane (29). FAK is a crucial signaling component activated by numerous stimuli, including growth factor receptors (epidermal and vascular endothelial growth factor receptors) and integrins, in order to regulate proliferation, survival and motility in normal cells as well as tumor cells (18). Breast cancer models have been employed to evaluate the role of FAK in regulating tumorigenic and metastatic properties (30). In addition, a study in human and mouse melanoma cell lines indicated that doxycycline inhibited adhesion and migration through downregulating the FAK signaling pathway (11). Furthermore, FAK signaling has been critically implicated in the generation of gelatinases and subsequent tumor invasion (31). However, it remained to be elucidated whether doxycycline exerts these effects on leukemia cells.

Acute leukemia is a hematopoietic malignancy that is widely circulated from its onset and may be regarded as a prototype of metastatic cancer (13). A previous study demonstrated that expression of FAK in leukemia was associated with enhanced blast migration and poor prognosis (16). Expression of gelatinases was also reported to have an essential role in the invasive capacity of AML and chronic myeloid leukemia, with emerging evidence suggesting that expression of these molecules may be mediated through the FAK/phosphoinositide 3-kinase (PI-3K)/extracellular signal-regulated kinase (ERK) signaling pathways (16,32,33).

The present study investigated the effects of doxycycline on the invasiveness of two myelogenous leukemia cell lines, KG1a and K562, as well as examined the role of the FAK signaling pathway and its influence on gelatinases in these effects. FAK is known to typically activate the migration of leukemic cells through the formation of integrin-dependent focal adhesions; in addition, β1-integrin (CD29) has been reported to be expressed by the KG1a and K562 cell lines (34,35). Therefore, it was hypothesized that treatment with a blocking anti-β1-integrin-Ab may inhibit migration of leukemic cells at the levels of transcription, translation and phosphorylation. In the present study, KG1a and K562 cells were treated with 100 ng/ml anti-β1-integrin-Ab for 24 h. As expected, the anti-β1-integrin-Ab potently decreased migration of the leukemic cells in Matrigel^®^ invasion assays. In addition, although mRNA levels of MMP-2 were significantly decreased in KG1a cells, MMP-9 mRNA levels were unchanged following treatment with anti-β1-integrin-Ab; these results were comparable to the effects of doxycycline. However, mRNA levels of MMP-2, MMP-9 and FAK remained stable in K562 cells following doxycycline or anti-β1-integrin-Ab. Furthermore, at the protein level, the expression levels of FAK and MMP-2 as well as the phosphorylation of Tyr397 and Tyr925 were potently decreased by anti-β1-integrin-Ab treatment of KG1a cells. These results were comparable to the effects of doxycycline in KG1a. In K562 cells, anti-β1-integrin-Ab treatment inhibited the expression of MMP-2 and phosphorylation of Tyr576 and Tyr925.

Cell migration is essential to tumor invasion and metastasis; therefore, the present study focused on the capacity of doxycycline to attenuate the migration of leukemic cells through inhibiting the FAK signaling pathway. FAK activation and degradation of the ECM have important roles in cell migration (36); therefore, it was hypothesized that doxycycline-mediated reduction of FAK and gelatinases may lead to decreased cell invasiveness. This hypothesis was tested in the present study using Matrigel^®^ invasion assays, which demonstrated that exposure of KG1a and K562 leukemic cells to doxycycline decreased their invasive capacity. mRNA levels of FAK and the gelatinases (MMP-2 and MMP-9) were almost unchanged in K562 cells following doxycycline treatment; however, identical treatment of KG1a cells resulted in significantly decreased MMP-2 mRNA levels, while those of MMP-9 were increased. These data indicated that doxycycline exhibited no significant effect on FAK transcription in leukemic cells and demonstrated that the different leukemic cell lines had various sensitivities to doxycycline at the level of gelatinase transcription. These observations suggested that, paradoxically, transcription of FAK and gelatinases may not be essential for leukemic cell migration.

Phosphorylation, in particular tyrosine phosphorylation, is important for kinase activity and represents another mode of FAK regulation. FAK contains several tyrosine residues, including Tyr397, 407, 576, 577, 861 and 925, which are able to be phosphorylated (12). Tyr397 autophosphorylation is a key event in FAK activation, which results in the generation of a Src-homology-2 (SH2) binding site for Src (37). Following upstream activation of Src, the Tyr576/577 site of FAK is phosphorylated, which promotes maximal FAK catalytic activation (18). Src was also reported to phosphorylate the downstream effector FAK at Y407, 861 and 925. Phosphorylation of FAK by Src regulates its kinase activity and localization as well as its cellular motility and invasion (38). Of note, phosphorylation of the FAK C-terminal Tyr925 has been associated with Src-induced focal contact dynamics. In addition, phosphorylation of Tyr925 was reported to induce the generation of growth factor receptor-bound protein 2 (GRB2) binding site for GRB2, which activated the Rasmitogen-activated protein kinase signaling cascade to promote focal contact turnover and contribute to cancer cell migration (39).

There is evidence to suggest that FAK-mediated signaling via Ras-related C3 botulinum toxin substrate 1 and Jun N-terminal kinases as well as FAK/PI-3K/ERK signaling may contribute to the expression of gelatinases and FAK-enhanced motility (40). Activation of MMPs is known to promote matrix proteolysis, leading to the extracellular release of integrin-matrix contacts, thereby facilitating focal contact remodeling and cell migration (41).

The present study demonstrated that the invasion ability of KG1a and K562 cells was inhibited by doxycycline; however, these results also indicated the existence of two different mechanisms underlying this effect in these leukemic cell lines. In KG1a cells, decreased expression of FAK following doxycycline treatment resulted in the inhibition of Tyr397 and Tyr925 phosphorylation, which subsequently led to downregulation of gelatinase expression, attenuation of focal contact turnover and blockade of ECM degradation. In K562 cells, doxycycline had no effect on expression of FAK and phosphorylation of Tyr397; however, doxycycline was able to downregulate Tyr576/577 and Tyr925 phosphorylation, ultimately leading to decreased focal contact remodeling and expression of MMP-2.

In the present study, analogous results to those of doxycy-cline were observed following treatment of KG1a and K562 cells with anti-β1-integrin-Ab, which attenuated migration in these leukemic cells through inhibiting the expression and phosphorylation of FAK. These results suggested that doxycycline exerted its antimigratory effects through the FAK signaling pathway. In addition, a previous study demonstrated that dysfunction of β1-integrin and FAK in K562 cells lead to abnormal adhesive characteristics (42). It was therefore proposed that disruption of aberrant β1-integrin-mediated signaling by doxycycline restored normal adhesion mechanisms, which further indicated that the integrin-FAK signaling pathway may be a potential target of doxycycline.

In conclusion, the results of the present study demonstrated that doxycycline exerted a potent activity against the migration of leukemic cells *in vitro*. In addition to downregulation of FAK and its phosphorylation, the inhibition of downstream gelati-nases was suggested to be involved in doxycycline-mediated inhibition of migration. These results therefore indicated the potential of doxycycline to attenuate the migration of leukemic cells through inhibiting the FAK signaling pathway.

## Figures and Tables

**Table I:** Effects of doxycycline and anti-β1 integrin-Ab on the invasiveness of KG1a and K562 human leukemia cell lines.

Group	No. of migrated leukemia cells	
	KG1a	K562
Control	25.00±3.91	24.25±4.57
Doxycycline	11.75±4.43^a^	7.00±2.16^a^
Anti-β1 integrin-Ab	5.00±1.00^a^	3.50±1.29^a^

Values are presented as the mean ± standard deviation.

a P<0.05 vs. control group. Ab, antibodies.
